# Establishment of a Hyperacute Rejection Model of ABO-Incompatible Renal Transplantation in Nonhuman Primates

**DOI:** 10.3389/fimmu.2021.807604

**Published:** 2021-12-14

**Authors:** Junxiang Wang, Hao Feng, Chi Zhang, Shan Zhong, Lu Wang, Lan Zhu, Song Chen, Gang Chen

**Affiliations:** ^1^ Institute of Organ Transplantation, Tongji Hospital, Tongji Medical College, Huazhong University of Science and Technology, Wuhan, China; ^2^ Key Laboratory of Organ Transplantation, Ministry of Education, Wuhan, China; ^3^ National Health Commission (NHC) Key Laboratory of Organ Transplantation, Wuhan, China; ^4^ Key Laboratory of Organ Transplantation, Chinese Academy of Medical Sciences, Wuhan, China

**Keywords:** ABO-incompatible renal transplantation, hyperacute rejection, blood group antibody, flow cytometry, non-human primate (NHP)

## Abstract

The establishment of a hyperacute rejection (HAR) model of ABO-incompatible kidney transplantation (ABOi-KTx) in nonhuman primates is of great significance for the study of the relevant clinical pathophysiological processes and related interventions in ABOi-KTx. In this study, blood group B cynomolgus monkeys were presensitized with synthetic blood group A-antigen conjugated to keyhole limpet hemocyanin (A-KLH) to boost circulating anti-A antibody levels. The serum anti-A antibody levels were measured by flow cytometry using type A human reagent red blood cells (RBCs) or monkey primary renal tubular epithelial cells (RTECs) as target cells. ABOi-KTx was performed in type B monkeys using type A monkeys as donors. After 14 days of A-KLH sensitization, 12 of 16 (75%) type B monkeys had significantly elevated anti-A antibody levels. We found that in order to avoid irregular results in the detection of blood group antibodies by flow cytometry, it was more effective to use RTECs rather than RBCs as target cells. In the absence of presensitization, ABOi-KTx in three monkeys with relatively high levels of natural anti-A antibodies did not produce HAR. However, when four Type B monkeys with significantly increased anti-A antibodies after presensitization were randomly selected as recipients for ABOi-KTx, the allografts in all four monkeys developed HAR with typical pathologic characteristics. Thus, we have successfully established a monkey model of HAR in ABOi-KTx *via* blood group antigen presensitization, which will be helpful for the further study of rejection, accommodation, and clinical intervention in ABOi-KTx.

## Introduction

ABO-incompatible kidney transplantation (ABOi-KTx) is usually contraindicated because of the risk of hyperacute rejection (HAR) mediated by naturally occurring antibodies specific to donor blood group antigens (1). Nonetheless, unremitting donor organ shortages fuel attempts to cross the ABO barrier. ABOi-KTx has been shown to be safe, with acceptable long-term outcomes, when pretransplant desensitization strategies are used to reduce the accumulation of ABO blood group antibodies and B cells/plasma cells (2–4). Although some studies evaluating patient and graft outcome after ABOi-KTx have not shown any striking differences when compared with living donor ABO-compatible kidney transplantation (ABOc-KTx) (5–7), larger registry data have yielded conflicting results (8–10). Within the first 5 years after transplantation, ABOi-KTx is associated with a higher risk of mortality, loss of kidney grafts, infectious complications, and acute antibody-mediated rejection (AAMR) than is ABOc-KTx (10).

In order to further elucidate the clinical pathophysiological processes involved in ABOi-KTx and to conduct related intervention studies, it is important to establish a clinically relevant nonhuman primate study model. Like humans, nonhuman primates express the ABH-specific antigens of the ABO histo-blood group system on the vascular endothelium, epithelial cells, and exocrine secretions (11). However, these ABH antigens are absent from or only weakly expressed on erythrocytes of both Old and New World monkeys (11, 12). Baboons and macaques, two typical representatives of Old World monkeys, are frequently used in experimental transplantation research because of their physical traits and availability. Since the sera of baboons and macaques regularly contain naturally occurring anti-A and/or anti-B antibodies (11, 13), the A and/or B antigens expressed on vascular endothelial and epithelial cells can be targets for immune rejection by a recipient (11, 14, 15), and they may exhibit a process of ABO-incompatible rejection similar to that seen in humans.

The incidence of HAR has been estimated to be approximately 66% in humans that have received ABOi-KTx without any pretransplant desensitization treatment (16, 17). In contrast, previous studies have shown that ABO incompatibility does not cause HAR in primate kidney transplant models (18–20), whereas it leads to early HAR in approximately 33% of baboon heart transplants (15, 21). In order to achieve a high incidence of HAR, a presensitization strategy has been used to increase the levels of anti-A or anti-B antibodies in baboon recipients prior to ABO-incompatible cardiac transplantation (22), but there appear to be no data on successful HAR in a primate renal transplantation model between ABO-incompatible pairs.

Therefore, in the present study we have presensitized blood type B recipient cynomolgus monkeys with synthetic blood group A antigen to establish a reliable nonhuman ABOi-KTx model in which all kidney allografts from blood type A donors undergo HAR. In addition, we have developed an improved flow cytometry technique for the detection of blood group antibody. We show that by using monkey primary renal tubular epithelial cells (RTECs) expressing blood group antigen A rather than red blood cells (RBCs) as target cells, the serum levels of anti-blood group A antibodies can more accurately be evaluated by flow cytometry.

## Materials and Methods

### Animals

Outbred male cynomolgus monkeys (*Macaca fascicularis*, n=25), seronegative for simian immunodeficiency virus and herpes B virus, were obtained from Guangzhou Landao Biotechnology Corporation & South China Primates Research Center (Guangzhou, China). The monkeys were between 3 and 7 years old and weighed 3-8 kg. They were housed in the primate facility at the Experimental Animal Center of Tongji Medical College according to the University’s Research Animal Resources guidelines. All animals underwent prospective ABO blood group typing using a previously described technique (13, 23). Since blood type B is more common than type A in cynomolgus monkeys (12, 13), 21 monkeys typed as B were chosen as potential transplant recipients, and 4 monkeys typed as A served as donors. All animal studies were approved and performed according to the guidelines of the Tongji Medical College ethical committee for animal experimentation. All of the experiments were performed under the guidelines of Tongji animal use regulations and approved by the Institutional Animal Care and Use Committee (IACUC) at the Tongji Medical College, Huazhong University of Science and Technology.

### A-KLH Presensitization

Specific blood group substance A was synthesized and conjugated to keyhole limpet hemocyanin (A-KLH, Sigma, MO, USA) in Chembiomed Ltd. (Edmonton, Alberta, CAN). A-KLH (2 mg) emulsified in 2 ml Freund’s adjuvant was injected subcutaneously in the back of each potential recipient monkey about 2 weeks before kidney transplantation. The goal of the A-KLH immunization program was to raise the anti-A antibody level in the potential monkey recipients (22).

### Sera Preparations

All sera were prepared as we have previously described (13, 23): Venous blood was collected from the monkeys into separation gel vacuum tubes for preparing serum, and 1 ml of serum collected from each monkey was pre-absorbed on human type O red blood cells (RBCs, Shanghai Blood/Biomedical Co. Ltd., Shanghai, CHN) to eliminate non-specific binding from anti-human heteroagglutinins.

### Detection of Anti-A Antibody Levels by Flow Cytometry Using Human Type A RBCs

The amount of anti-A antibodies in the sera of the potential recipients was evaluated by measuring the binding of antibody to human type A1 reagent RBCs (Shanghai Blood/Biomedical Co., Ltd, Shanghai, CHN). In brief, serially diluted serum (50 μl) was incubated with 50 μl of human type A1 RBCs (1×10^7^/ml) for 30 min at 4°C and subsequently washed twice in FACS buffer (phosphate-buffered saline containing 0.01% sodium azide and 1% bovine serum albumin). Binding of anti-A antibodies were measured by indirect flow cytometry using secondary FITC-labeled goat anti-human IgG or IgM antibody (1:50, Zhongshan Biotechnology Co. LTD, Beijing, China). A minimum of 10,000 events were acquired on the flow cytometer (BD FACSAria Flow Cytometer, BD Biosciences, CA, USA). To evaluate anti-A antibody levels, the geometric mean fluorescence intensity (Gmean) of the cells obtained for each test serum was compared with that produced by the negative controls (cells incubated with medium instead of test serum). The ratio of relative mean fluorescence (RMF), defined as the ratio of the Gmean of the test serum to the Gmean of the negative control, was used to determine the degree of antibody binding to the target cells (24).

### Isolation and Culture of Monkey RTECs

After nephrectomy, fresh whole kidneys were harvested from monkeys typed as A in another project and flushed *via* the renal artery with 50 ml of ice-cold complete RPMI 1640 (Hyclone, USA) containing fetal bovine serum (10% v/v; Hyclone, USA), penicillin (100 U/ml), and streptomycin (100 μg/ml). After the kidney capsule was removed, the cortex was separated from the medulla, trimmed, and minced into approximately 1-mm^2^ pieces. The fragments were then incubated with complete RPMI 1640 containing collagenase/dispase (1 mg/mI; Roche, Germany) at 37°C in a gently shaking water bath (70 rpm). After 30 min, DNaseI (7.5U/ml; Fermentas, Canada) and MgCl_2_ (5 mM) were added to the mixture and incubated for another 30 min. The digested tissue mixture was then strained through a 150-μm cell strainer and centrifuged at 300 g for 5 min. Thereafter, the pelleted cells were resuspended in complete RPMI 1640 supplemented with sodium citrate (20 mM), overlaid on Ficoll (TBD, CHN), and centrifuged at 400 g for 20 min at 18°C (without braking). The viable cells were collected and washed twice with complete RPMI 1640 and then resuspended in complete Epithelial Cell Medium-animal (EpiCM-a; Sciencell, USA). The cell suspensions were placed in a 75-cm^2^ gelatin-coated flask and maintained in a cell incubator. After 48 h, the medium was replaced to remove tissue debris and non-adherent cells. Cells reached confluency and were first passaged at about 7 days. All experiments were conducted on cells from passage 7-10.

### Identification, Cryopreservation, and Resuscitation of RTECs

For flow cytometric characterization, 3×10^6^ cells were harvested and fixed with 1% paraformaldehyde in PBS for 15 min. After permeabilization with 0.1% Triton X-100 in PBS for 15 min at room temperature and blocking with PBS containing 2% bovine serum albumin for 30 min at 37°C, the cells were incubated with rabbit anti-keratin18 polyclonal antibodies (1:100, Proteintech Group Inc., CHN) or PBS (negative control) at 4°C overnight. The next day the cells were washed with PBS and incubated with secondary antibody (FITC-conjugated goat anti-rabbit IgG; 1:200, Zhongshan Biotechnology Co. LTD, Beijing, CHN) for 30 min at 37°C. After washing, cells were then analyzed on a flow cytometer (BD FACSAria Flow Cytometer, BD Biosciences, USA).

Adherent RTECs were detached from culture flasks with trypsin/EDTA (Life Technologies, USA), washed with complete DMEM/F12 medium (Hyclone, USA), and suspended in freshly prepared freezing medium containing fetal bovine serum (90%, v/v; Hyclone, USA) and dimethylsulfoxide (10%, v/v; Sigma-Aldrich, USA). All components used were pre-chilled at 4°C. The cell suspensions were then transferred to ice-cold 2ml cryovials and frozen at −80°C using a freezing container (Nalgene, USA). After being kept overnight at −80°C, the cryovials were transferred to a liquid nitrogen tank for long-term storage. Before use, the cell suspensions that had been stored at -80°C were thawed rapidly in a water bath at 37°C and washed twice.

### Detection of A Antigen Expression on Monkey Type A RTECs and Human Type A RBCs by Flow Cytometry

The expression of the A antigen on monkey type A RTECs or human type A1 reagent RBCs was determined by flow cytometry. In brief, 100 μl (2 × 10^5^) of RTECs or 100 μl (1 × 10^6^) of RBCs were incubated with 100 μl of 1:10 primary monoclonal antibody specific for either the A antigen or the B antigen (negative control) (Changchun Institute of Biological Products, Jilin, CHN) for 30 min at 4°C. After being washed with phosphate-buffered saline, cells were incubated with 10 μl of 1:100 phycoerythrin-labeled goat anti-mouse IgM (eBioscience, USA) for 30 min at 4°C. After another wash, the cells were analyzed on a flow cytometer (BD FACSAria Flow Cytometer, BD Biosciences, USA).

### Kidney Transplantation

Heterotopic kidney allotransplantation with bilateral nephrectomy was performed as described previously (24). In brief, following perfusion with histidine-tryptophan-ketoglutarate solution (HTK solution, Germany), the left kidney was removed from the type A donor monkey and kept in ice-cold HTK solution until it was transplanted into the type B recipient monkey. The renal graft was implanted heterotopically into the right abdomen of the recipient monkey, with end-to-side anastomoses of the renal artery to the recipient aorta and the renal vein to the recipient vena cava. After reperfusion, the donor ureter was implanted into the recipient bladder with interrupted 7-0 polydioxanone (PDS) sutures. Both native kidneys were removed from the recipient during the transplant surgery.

### Post-Transplant Immunosuppression

Monkey recipients with no evidence of graft HAR received a maintenance immunosuppressive therapy consisting of hypodermic CsA (Novartis) to maintain a trough level between 300 ng/mL and 600 ng/mL, oral MMF (Cellcept) at 50 mg/kg/day, and oral prednisone at 1 mg/kg/day.

### Postoperative Studies

Recipients were checked twice a day to evaluate fluid balance, nutritional intake, behavior, and general condition. Body weight was measured daily for the first 7 days, then three times weekly. Blood was collected on the day before the renal transplant surgery and on days 1, 3, 7, 10 and 15, then weekly thereafter for complete blood counts and differentials, glucose, protein, electrolytes and creatinine levels. The study endpoint was defined as (1) evidence of severe of renal failure (serum creatinine > 700 μmol/L); (2) clinical deterioration from other causes believed to be irreversible, or (3) unexpected death. Monkeys meeting conditions 1 or 2 were humanely euthanized. In all cases, a full necropsy was performed to confirm the cause of death, and tissue samples were collected for histopathologic analysis.

### Histopathology and Immunohistochemistry

For light microscopy, 10% buffered formalin-fixed, paraffin-embedded material was processed and stained with hematoxylin and eosin (HE). Immunofluorescent examinations for IgG, IgM, C3c, and C5b-9 were performed on frozen material (-80°C) as previously reported (24). Immunohistochemical staining for CD3, CD20, and CD68 was performed on paraffin-embedded material using immunoperoxidase methods as described previously (24).

### Statistical Analysis

The statistical differences between two groups were analyzed by an unpaired Student’s t-test or a Mann-Whitney Rank Sum Test (SPSS 10.0; SPSS, Cary, NC). Animal survival differences between groups were determined using the log-rank test (GraphPad Prism 6.0; GraphPad Software; GraphPad, Bethesda, MD). A value of *p*<0.05 was considered significant.

## Results

### ABOi-KTx in Monkeys Does Not Result in HAR Without Presensitization

**Figure 1:**
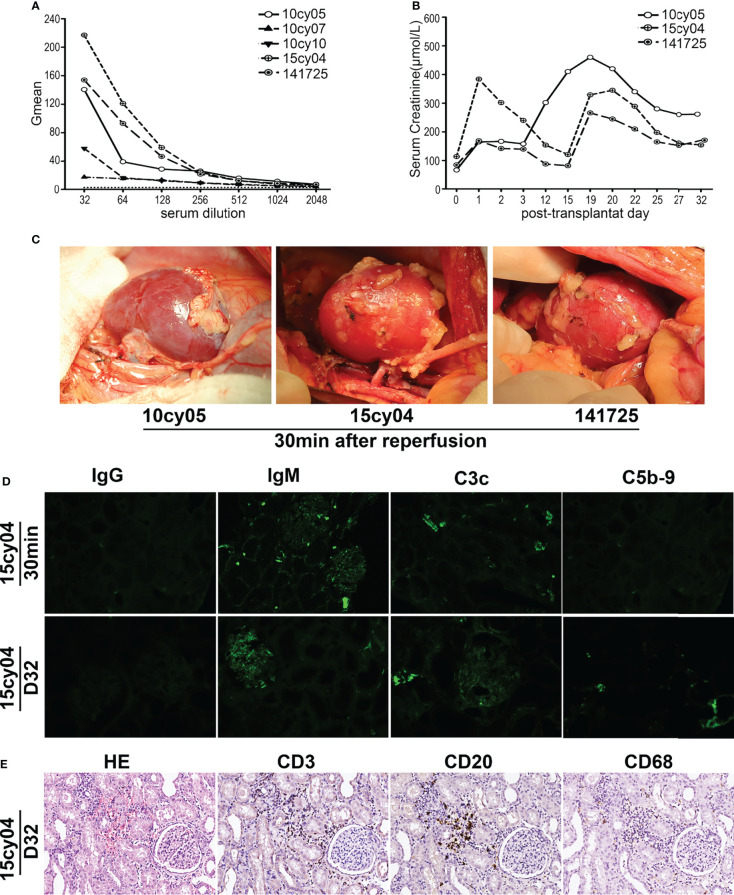
Without A-KLH presensitization, ABOi-KTx in monkeys does not result in HAR. **(A)** Natural anti-A IgM levels were tested in serially diluted sera by flow cytometry using type A reagent RBCs as target cells in five monkeys (10cy05, 10cy07, 10cy10, 15cy04 and 147125). **(B)** Three monkeys (10cy05, 15cy04 and 141725) with relatively high levels of natural anti-A IgM, were selected for ABOi-KTx. Changes in serum creatinine levels after ABOi-KTx in the three recipient monkeys are shown. **(C)** The color of the renal grafts were almost completely normal after blood reperfusion during the operation in the three monkeys. **(D)** A typical immunofluorescent staining (monkey 15cy04) for IgG, IgM, C3c, and C5b-9 on the allograft biopsy tissues (30min after blood reperfusion) and autopsy tissues (day 32). **(E)** A typical hematoxylin-eosin (HE 20×) staining and immunohistochemical staining (20×) for CD3, CD20, and CD68 (day 32) (monkey 15cy04).

First, we sought to determine whether ABOi-KTx in monkeys would result in HAR in the absence of presensitization. Among 18 potential recipient monkeys with blood group B, we selected three monkeys with relatively high levels of natural anti-A IgM to receive transplants by ABOi-KTx from type A monkey donors, without having undergone any presensitization (**Figure 1A** and **Table 1**). The transplanted kidneys were observed for at least 1 hr after blood reperfusion during the operation, and their color were almost completely normal (**Figure 1C**). With the CsA-based immunosuppressive maintenance therapy, all three renal allografts survived more than 30 days, despite some fluctuation in serum creatinine levels during follow-up (**Figure 1B**). Immunofluorescent examination of the biopsy tissue of the allografts at 30 min after blood reperfusion showed weakly positive staining for IgM and C3c deposition and almost no deposition of IgG and C5b-9 (**Figure 1D**). The recipient monkeys were euthanized on day 31, 32 and 33, respectively. Pathologic analysis showed weak antibody-mediated rejection with mild deposition of IgM, C3c and C5b-9 in the grafts (**Figure 1D**). In addition, mild to moderate infiltration of T cells (CD3^+^), B cells (CD20^+^) and neutrophils/macrophages (CD68^+^) were seen in the grafts, indicating that cell-mediated rejection had developed in the grafts (**Figure 1E**).

**Table 1:** Pre- and postimmunization anti-A IgM/IgG antibody levels measured by flow cytometry using human red blood cells as target cells.

Monkey	Anti-A IgM		Peak	Irregular	Anti-A IgG		Peak	Irregular
	Preimmunization	Postimmunization (D14)			Preimmunization	Postimmunization (D14)		
	RMF(1:32 sera)	RMF(1:32 sera)	RMF	RMF	RMF(1:32 sera)	RMF(1:32 sera)	RMF	RMF
10cy05	55.76	No immunization	1:32	none	1.55	No immunization	1:32	none
10cy10	22.98	No immunization	1:32	none	2.32	No immunization	1:32	none
10cy07	6.98	No immunization	1:32	none	6.66	No immunization	1:256	none
15cy04	86.31	No immunization	1:32	none	2.35	No immunization	1:32	none
141725	61.24	No immunization	1:32	none	4.41	No immunization	1:32	none
08cy08	9.62	536.96	1:32	1:128	1.14	21.08	1:256	none
07cy05	3.62	478.21	1:32	1:128	1.29	13.86	1:64	1:128
09cy14	7.45	467.21	1:64	1:128	1.54	34.73	1:256	1:512
09cy07	7.76	302.87	1:64	1:256	2.57	27.02	1:2048	none
10cy16	11.12	270.63	1:32	1:64	1.71	10.66	1:1024	none
10cy19	7.34	85.30	1:32	1:128	1.21	23.60	1:256	1:512
11cy03	9.18	87.44	1:32	1:64	0.94	2.83	1:8192	none
11cy02	4.86	70.01	1:32	1:64	3.48	37.61	1:2048	none
11cy04	8.53	60.22	1:32	1:64	1.25	14.30	1:32	1:64
09cy15	9.45	29.22	1:32	none	1.29	22.22	1:32	1:64
09cy01	6.24	4.59	1:32	none	2.59	3.12	1:32	none
10cy14	2.51	2.87	1:32	none	2.48	2.51	1:32	none
10cy20	2.77	2.66	1:32	none	2.45	2.63	1:32	none

RMF, relative mean fluorescence.

### Anti-A Antibody Levels Are Remarkably Increased by A-KLH Presensitization

In the early part of this study, we measured anti-A antibody levels in 18 type B monkeys by flow cytometry using type A human reagent RBCs as target cells; the RMF of anti-A IgM in serum diluted 1:32 was used as the reference value for the antibody level for inter-sample comparisons. As shown in **Table 1**, the baseline RMF range of natural anti-A IgM and IgG in the 13 cynomolgus monkeys was 2.51 to 11.12 and 0.94 to 3.48, respectively. After 14 days of A-KLH sensitization, the RMF of both anti-A IgM and IgG was significantly increased in 10 of the 13 monkeys, ranging from 29.22 to 536.96 and 2.83 to 37.61(*p*<0.001, vs. baseline), respectively. The other 3 monkeys did not respond significantly to A-KLH presensitization (p>0.05 vs. baseline).

### When Human RBCs Are Used as Target Cells, Irregular Results Can Occur in Flow Cytometry Analysis of Serially Diluted Serum Samples

**Figure 2:**
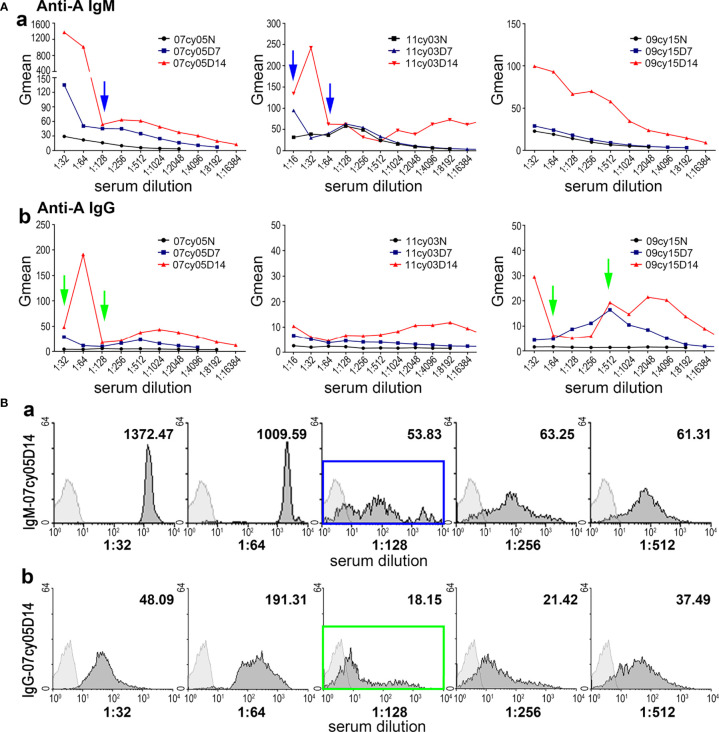
Measuring anti-A antibody by flow cytometry using human type A RBCs as target cells can produce irregular results. **(A)** The anti-A IgM **(a)** and IgG **(b)** levels before (N) and after A-KLH presensitization (day7 and day 14) in three monkeys (07cy05, 11cy03, and 09cy15), determined in serially diluted sera by flow cytometry using type A human reagent RBCs as target cells. Confusing and unpredictable results for IgM and IgG are indicated by the blue and green arrows, respectively. **(B)** Flow cytometric results for anti-A IgM **(a)** and IgG **(b)** in a representative monkey (07cy05) 14 days after A-KLH presensitization. Irregular results are indicated by the blue and green squares.

In the first five monkeys used in this study (10cy05, 10cy10, 10cy07, 15cy04 and 141725), we assessed the baseline natural anti-A IgM levels in serially diluted sera by flow cytometry using type A human reagent RBCs as target cells. The curves of the Gmean results were relatively regular and predictable (**Figure 1A**). However, in most monkeys after A-KLH presensitization, the Gmean results for either IgM or IgG, or both, were confusing and unpredictable, especially in the remaining 10 monkeys whose levels of induced anti-IgM antibody were high (**Table 1** and **Figure 2**). For example, at 14 days after A-KLH presensitization, the Gmean value was 1009.59 in a 1:64 dilution of serum from monkey 07cy05, but it dropped abruptly to 53.83 at a 1:128 dilution, and the peak graphics of flow cytometry were also confusing (**Figure 2**, blue arrow and square). The same problem occurred in the flow cytometric determination of anti-A IgG levels when reagent RBCs were used as target cells (**Figure 2**, green arrow and square).

### Monkey RTECs Express Blood Group Antigens Stably and Can Replace Human RBCs as Target Cells for Flow Cytometry

**Figure 3:**
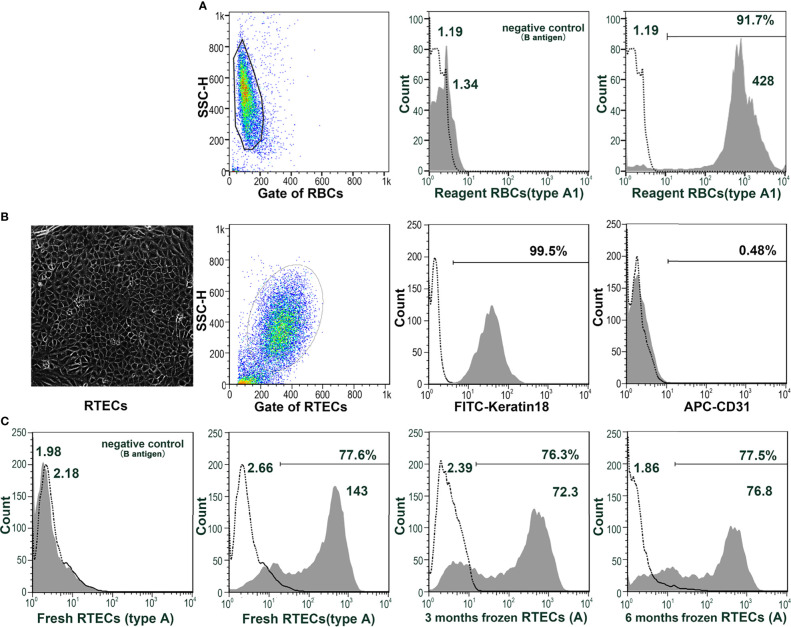
Monkey RTECs stably express blood group antigens. **(A)** Blood group A antigen (not B antigen) is expressed on human type A1 reagent RBCs. **(B)** RTECs were isolated from the cortices of type A monkey kidneys and identified as keratin18^+^CD31^-^. **(C)** Blood group A antigen (not B antigen) can be stably expressed on type A RTECs, either fresh or frozen (for 3 or 6 months).

**Figure 4:**
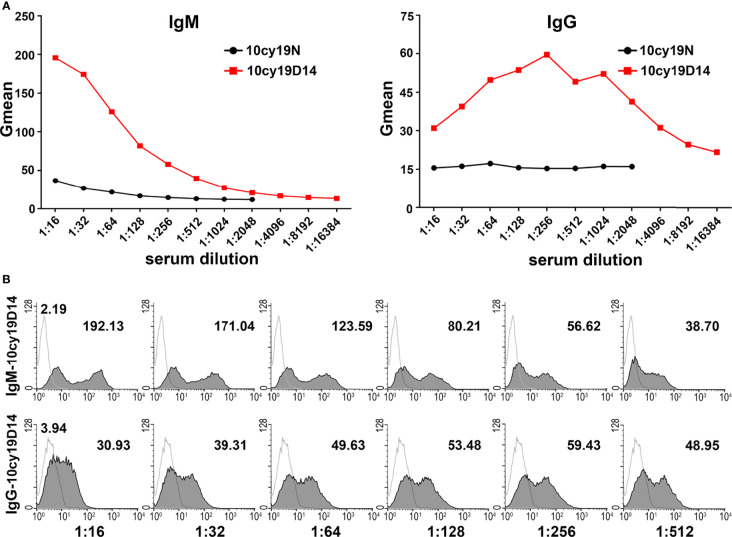
Anti-A antibodies can be effectively detected by using monkey type A RTECs as target cells in flow cytometry. **(A)** Anti-A IgM and IgG levels before (N) and after A-KLH presensitization (day 14) in serially diluted sera as examined by flow cytometry using type A monkey RTECs as target cells (monkey 10cy19). **(B)** Flow cytometric results for anti-A IgM and IgG in monkey 10cy19 at 14 days after A-KLH presensitization. The flow cytometric peak graphics here are reasonable and predictable.

RTECs were isolated from the cortex of type A monkey kidneys and identified as keratin18^+^CD31^-^ (**Figures 3A, B**). Even after cryopreservation for up to 6 months, blood group A antigen was stably expressed on the type A RTECs (**Figure 3C**), making RTECs potential target cells for flow cytometry. A typical flow cytometric result (monkey 10cy19) using RTECs as target cells is shown in **Figure 4**: We found that the anti-A antibody levels before and after A-KLH presensitization showed an obvious stratified distribution, without irregular peaks or cliffs. When we tested the anti-A antibody levels in serum samples from a total of 16 type B monkeys (including the 13 monkeys described above and 3 additional monkeys) by using this modified method for flow cytometry, we found no instances of irregular RMF. As shown in **Table 2**, the baseline RMF values for anti-A IgM and IgG ranged from 2.6 to 26.7 and from 1.68 to 3.89, respectively, in the 1:16 diluted sera of the 16 cynomolgus monkeys. Among the 16 monkeys, 12 (75%) showed a significant increase in the RMF for both induced anti-A IgM and IgG after A-KLH presensitization, ranging from 36.83 to 144.83 and 4.66 to 15.39 (p<0.001, vs. baseline), respectively. The other 4 monkeys showed no significant response to A-KLH presensitization (p>0.05) (**Table 2**).

**Table 2:** Pre- and postimmunization anti-A IgM and IgG levels measured by flow cytometry using monkey renal tubular epithelial cells as target cells.

Group	Monkey	Anti-A IgM		Anti-A IgG		ABOi-KTx
		Preimmunization	Postimmunization (D14)	Preimmunization	Postimmunization (D14)	
		RMF (1:16 sera)	RMF (1:16 sera)	RMF (1:16 sera)	RMF (1:16 sera)	
1	07cy05	4.55	116.16	1.88	10.61	YES
	09cy15	4.18	97.39	2.24	15.39	
	11cy03	26.70	119.26	3.21	8.64	
	12cy03	5.96	43.28	1.94	4.66	
2	08cy08	18.13	98.31	2.29	11.44	NO
	09cy07	2.60	122.93	3.13	9.82	
	09cy14	24.62	131.10	2.75	8.80	
	10cy16	21.91	50.73	2.75	5.22	
	10cy19	17.55	87.73	3.89	7.85	
	11cy02	4.40	129.10	2.20	6.65	
	11cy04	9.77	144.83	2.48	8.46	
	13cy06	13.72	36.83	1.97	5.10	
3	09cy01	5.53	6.45	2.25	2.82	NO
	10cy14	2.99	3.29	1.81	2.06	
	10cy20	3.12	3.62	1.83	1.99	
	13cy04	7.16	6.28	1.68	1.59	

RMF, relative mean fluorescence; ABOi-KTx, ABO-incompatible kidney transplantation.

### Type A Renal Allografts Show HAR in Type B Recipient Monkeys Exhibiting Significantly Increased Anti-A Antibody Levels After Presensitization

**Figure 5:**
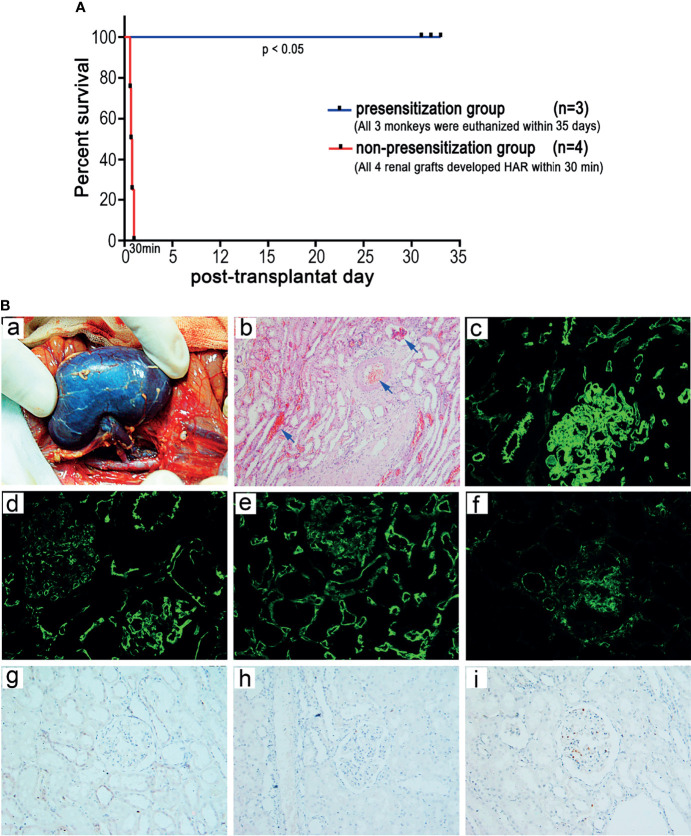
Establishment of a HAR model of ABOi-KTx in nonhuman primates**. (A)** Renal allograft survival after ABOi-KTx in the recipient monkeys with or without A-KLH presensitization (*P*<0.05, presensitization group vs. non-presensitization group). **(B)** Representative gross and pathological changes in a typical renal graft with severe HAR after ABOi-KTx (monkey 07cy05). (a) Gross findings indicating severe HAR in the terminal graft. (b) HE staining of the terminal graft showing severe interstitial hemorrhage, necrosis, and intravascular thrombosis (blue arrows). (c-f) Immunofluorescent staining of the terminal graft showing massive deposition of IgM (c), C3c (d), and C5b-9 (e) and moderate deposition of IgG (f). (g-i) Immunohistochemical analysis showing no CD3^+^ (g) or CD20^+^ (h) cell infiltration and minimal CD68^+^ cell infiltration (i).

To determine whether HAR can occur in ABOi-KTx in nonhuman primates after blood group antigen presensitization, we randomly selected 4 of the 12 type B monkeys with significantly increased anti-A antibody levels after presensitization as recipients to receive kidney transplantation from type A donors (**Table 2**). All four renal grafts (100%) developed HAR within 30 min after blood reperfusion (**Figure 5A**), with a general dark-purple appearance (**Figure 5B-a**). Graft pathology showed typical features of HAR, characterized by severe interstitial hemorrhage; necrosis; intravascular thrombosis (**Figure 5B-b**, blue arrow); massive deposition of IgM, C3c, and C5b-9 (**Figure 5B-c,d,e**); and moderate deposition of IgG (**Figure 5B-f**). Immunohistochemical analysis showed no T-cell (CD3^+^) or B-cell (CD20^+^) infiltration and minimal neutrophil/macrophage (CD68^+^) infiltration (**Figure 5B-g,h,i**).

## Discussion

Given the serious shortage of donor organs, living relative kidney transplantation has become more and more widespread. However, ABO incompatibility between related donors and recipients is rather common. If ABOi-KTx is performed, HAR or AAMR may occur, which will expose the transplant to a great risk of failure (1, 4, 7, 8). As a result, these patients often do not get the chance to receive living relative kidney transplantation and are forced to wait for a long time for a transplant. The establishment of animal models of HAR in ABOi-KTx is of great significance for exploring effective preventive and treatment approaches for the incompatibility problem. However, to date we are unaware of any reports on the successful establishment of such animal models. In the present study, we have presensitized blood type B recipient monkeys with synthetic blood group A antigens and have successfully established a reliable HAR model of ABOi-KTx in cynomolgus monkeys.

Unlike the case in humans, and despite the occurrence of natural anti-blood group antibodies in the sera and expression of the target antigens in tissues, hyperacute rejection is rare following solid organ transplantation in nonhuman primates (15, 18–21). Cooper and coworkers have studied ABO-incompatible heart transplantation in baboons and report that only a small number of animals develop acute humoral rejection after transplantation, with most animals showing chronic humoral rejection because of the weak degree of humoral injury (21). In the present study, we performed ABOi-KTx in three recipient cynomolgus monkeys with relatively high baseline levels of natural antibodies against the donor blood group antigen. As a result, none of the renal grafts develop HAR. Given this scenario, it is difficult to establish hyperacute or acute humoral rejection models of ABO-incompatible transplantation directly in nonhuman primates.

Since the general levels of circulating blood group antibodies in nonhuman primates are significantly lower than in humans, they are not sufficient to cause HAR in ABO-incompatible transplantation in nonhuman primates. In order to establish the HAR model, the circulating blood group antibody level must be significantly elevated. It appears to be an effective strategy to use the injection of blood group antigens to induce nonhuman primates to produce a higher level of the corresponding blood group antibodies. Given the insufficient immunogenicity of the blood group antigens, we reasoned that conjugating the blood group antigens to KLH should greatly enhance the immunogenicity of the blood group antigens, stimulating the body to rapidly produce a large amount of induced antibody. This immunization strategy was first reported in an ABO-incompatible heart transplant model in baboons, but the details of the method were not provided (22). In the present study, we injected synthetic A-KLH emulsified in Freund’s adjuvant to presensitize 16 blood type B cynomolgus monkeys. As expected, the levels of anti-A antibodies were remarkably increased in 12 (75%) of the monkeys. To determine whether a HAR model could be established, we randomly selected 4 of these 12 monkeys as recipients to receive ABOi-KTx from type A donors and found that all the grafts (100%) developed typical HAR shortly after blood reperfusion. Therefore, we have demonstrated for the first time that the HAR model can be successfully established in ABO-incompatible kidney transplantation in monkeys.

For ABOi-KTx research, it is quite important to establish a stable and reliable measurement technique for determining blood group antibody levels. In the early stages of this study, we used a flow cytometric analysis employing human reagent RBCs as target cells as a semiquantitative method to monitor the levels of anti-blood group IgG and IgM in monkeys, as described previously (25, 26). Reasonable and explicable results were obtained by this method when the monkey sera contained low levels of natural blood group antibodies. However, when the anti-A antibody levels were significantly elevated after A-KLH presensitization, the detection results obtained using this method became confusing and unpredictable. We may be dealing here with artefacts related to erythrocyte agglutination caused by high levels of blood group antibodies. Therefore, we hypothesized that using other kind of cells as the target cells for flow cytometry, i.e., cells that can express blood group antigens and avoid agglutination, may produce more consistent results.

As a type of karyocyte, RTECs are larger in size and more stable than RBCs. The stable expression of blood group antigens and non-agglutination of these cells make RTECS ideal target cells for flow cytometry. With our modified method of flow cytometry with monkey RTECs as target cells, we were able to clearly determine the anti-A antibody levels in monkey sera before and after A-KLH presensitization. Based on our results, RMF>30 is the minimum IgM antibody threshold level that we recommend to use in generating HAR. In human kidneys, vascular endothelium is reported to express only A-subtype II, while tubular epithelium is reported to express A-subtypes II-III/IV (27). Due to the difference in A antigen expression between vascular endothelial cells and RTECs, which cells are more suitable as the target cells for detection of anti-A antibodies by flow cytometry is worth further study.

In conclusion, we have established a clinically relevant non-human primate animal model for studying HAR in ABOi-KTx. In addition, we have also established a novel flow cytometric semi-quantitative assay employing monkey RTECs as target cells that can be used to effectively and clearly measure blood group antibody levels. The results of our study will be useful for further studies of rejection, accommodation, and clinical intervention in ABOi-KTx.

## Data Availability Statement

The original contributions presented in the study are included in the article/supplementary material. Further inquiries can be directed to the corresponding authors.

## Ethics Statement

The animal study was reviewed and approved by Institutional Animal Care and Use Committee (IACUC) at the Tongji Medical College.

## Author Contributions

GC designed the experiments. JW, HF, CZ, and LW participated in the performance of the research and data analysis. GC, SC, SZ, and LZ performed the transplant surgery. SC and JW prepared the figures and wrote the article. GC critically revised the article. All authors contributed to the article and approved the submitted version.

## Funding

This study was supported by the National Natural Science Foundation of China (30872384) and the Major Scientific and Technological Project of Hainan province (ZDKJ2019009).

## Conflict of Interest

The authors declare that the research was conducted in the absence of any commercial or financial relationships that could be construed as a potential conflict of interest.

## Publisher’s Note

All claims expressed in this article are solely those of the authors and do not necessarily represent those of their affiliated organizations, or those of the publisher, the editors and the reviewers. Any product that may be evaluated in this article, or claim that may be made by its manufacturer, is not guaranteed or endorsed by the publisher.

## Abbreviations

AAMR, acute antibody mediated rejection; ABOc-KTx, ABO-compatible kidney transplantation; ABOi-KTx, ABO-incompatible kidney transplantation; Gmean, geometric mean fluorescence intensity; HAR, hyperacute rejection; KLH, keyhole limpet hemocyanin; RBCs, red blood cells; RMF, relative mean fluorescence; RTECs, renal tubular epithelial cells.
